# Monkeypox: A New Challenge for Global Health System?

**DOI:** 10.3390/life13061250

**Published:** 2023-05-25

**Authors:** Francesca Spirito, Agostino Guida, Vito Carlo Alberto Caponio, Lorenzo Lo Muzio

**Affiliations:** 1 Department of Clinical and Experimental Medicine, University of Foggia, Via Rovelli, 71122 Foggia, Italy; vitocarlo.caponio@unifg.it (V.C.A.C.); lorenzo.lomuzio@unifg.it (L.L.M.); 2 U.O.C. Odontostomatologia, A.O.R.N. “A. Cardarelli”, 80131 Naples, Italy; agostino.guida@aocardarelli.it; 3 Consorzio Interuniversitario Nazionale per la Bio-Oncologia, 66100 Chieti, Italy

**Keywords:** monkeypox, virus diseases, vaccine

## Abstract

The COVID-19 pandemic, starting in 2020, has presented a major challenge in terms of early diagnosis and the subsequent containment and management of severe cases. The spread of viruses such as monkeypox in non-endemic countries is now creating new difficulties for healthcare professionals. Proper case definition and clinical examination are crucial for the early identification of suspected cases. For this reason, we performed a review of the literature in order to report the first signs, which are useful for healthcare providers for early case identification. Since 2022 to date, 86,930 laboratory-confirmed cases and 1051 probable cases have been reported worldwide, and of these, 116 were fatal cases and, for the first time, most of the cases were registered in countries that have not historically reported monkeypox and that lack direct or immediate epidemiological links to areas of West or Central Africa where the disease is endemic. Patients with Monkeypox experience prodromal symptoms, such as fever, fatigue, headache, muscle aches, and a rash after an incubation period of 5–21 days. The disease is usually self-limiting within 2–4 weeks but can lead to complications, such as pneumonia, encephalitis, kidney injury, and myocarditis in children, pregnant individuals, and those with weakened immune systems. The case–fatality ratio is between 1 and 10%. Today, prevention campaigns and the control of human monkeypox are the best weapons to prevent infection and stop transmission. Prevention strategies, such as avoiding contact with sick or dead animals, and the proper preparation of all foods containing animal meat or parts, should be adopted. Furthermore, close contact with infected people or contaminated materials should be avoided to prevent human-to-human transmission.

## 1. Introduction

The monkeypox virus is a double-stranded DNA virus belonging to the *Poxviridae* family, genus *Orthopoxvirus* (the same genus as the variola virus that causes smallpox, and the vaccinia virus that causes cowpox), which was first identified in laboratory crab-eating Macaques (Asian Macaca Fascicularis monkeys) in Copenhagen in 1958 [1] by von Magnus during two outbreak of a vesicular eruptive disease in the summer and autumn of that year [2]. von Magnus succeeded in isolating the monkeypox virus from a cell culture of monkey kidney tissue and from the chorioallantoic membrane of chick embryos. About thirty cases of monkeys with monkeypox were reported [2] more than fifty days after their arrival by ship from Singapore. There were no deaths and no cases of monkey-to-human transmission. Not all of the exposed monkeys exhibited the illness. 

From the results of the studies on the phenotypic characteristics of the virus, it was possible to conclude that the virus identified by von Magnus belonged to the smallpox–vaccinia group of Poxviridae [1]. 

In 1970, the first case of monkeypox infection in humans was reported in a region of the Democratic Republic of the Congo. This first identification of human monkeypox as a human pathogen was registered in a 9-month-old child who was living in a region where smallpox had been eliminated in 1968 [3].

After this first case, many other cases have been reported. The most affected areas were the rural, rainforest regions of the Congo Basin, particularly in the Democratic Republic of the Congo, but the infection has spread, leading to an increase in the number of cases that have been recorded, especially from the central and western areas of the African continent.

With the eradication of smallpox in 1980, the administration of vaccines to prevent smallpox strongly reduced worldwide in the 1980s, at a time when cases of human monkeypox in Africa were decidedly less predominant and worrying than cases of smallpox before eradication [4,5,6]. Therefore, monkeypox has emerged as the most important Orthopoxvirus with regard to its possible impact on public health [7].

In the COVID-19 pandemic era, the ongoing monkeypox virus outbreak had a certain resonance because, since the beginning of 2022 to date, 86,930 laboratory-confirmed cases and 1051 probable cases have been reported worldwide, and of these, 116 were fatal cases, and for the first time, most of these cases were registered in countries that have not historically reported monkeypox and that lack direct or immediate epidemiological links to areas of West or Central Africa where the disease is endemic.

On 23 July 2022, the Director General of the World Health Organization (WHO), Tedros Adhanom Ghebreyesus, declared the outbreak of monkeypox a Public Health Emergency of International Concern (PHEIC), and to date, it is still classified as this considering what was deliberate during the last meeting of the International Health Regulations (IHR) Emergency committee [8]. Today, the global risk is assessed as moderate, but it varies according to location. Countries and regions with a higher risk are mainly located in West Europe and Northern America. The low-risk countries are located in Africa and South Asia. The risk is assessed as low in countries located in Latin America and Asia [9].

The aim of this literature review is to gather all of the useful and relevant information regarding the virus, its spread and transmission, clinical approaches, and the management of monkeypox disease in order to create a clear picture for healthcare personnel who are on the front line in pandemic containment.

## 2. Diffusion

Human monkeypox virus was identified for the first time in humans in 1970 in the Democratic Republic of the Congo [3]; after that, 11 African areas have been involved in the spread of the human monkeypox virus: Benin, Cameroon, the Central African Republic, the Democratic Republic of the Congo, Gabon, Cote d’Ivoire, Liberia, Nigeria, the Republic of Congo, Sierra Leone, and South Sudan [10,11]. Indeed, in 1996–1997 in the Democratic Republic of the Congo, several human cases of monkeypox with a lower case–fatality ratio and a higher attack rate than usual were reported [12]. The diversity in the manifestation, transmission, and spread of this particular outbreak of cases has been partly explained by the possibility of a simultaneous infection of chickenpox (a varicella virus, not an orthopoxvirus) and monkeypox. Nigeria has experienced a cluster of numerous cases since 2017, with over 500 suspected cases and over 200 confirmed cases and a case–fatality ratio of approximately 3% [13,14]. The first time monkeypox cases were reported outside of Africa was in 2003, when forty-seven confirmed and probable cases were recorded in the United States from six states: Illinois, Indiana, Kansas, Missouri, Ohio, and Wisconsin, which was likely due to the importation of small mammals from Ghana [15]. Since 2018, several monkeypox virus outbreaks have been reported in the UK, Singapore, and the US. Other outbreaks have been reported sporadically in the United Kingdom [16]. 

The current outbreak began on 7 May 2022, when a case of human monkeypox, with a positive history of travel links to Nigeria, was reported by the UK Health Security Agency [7]. On 14 May in the United Kingdom (UK), the UK Health Security Agency (UKHSA) recorded a familial cluster of two human monkeypox cases. The peculiarity lies in the fact that these cases did not have any kind of contact with cases that were related to travel to Africa, as was typically the case before 7 May 2002. Since then, many monkeypox cases have been reported to the WHO from several member states across different WHO regions. Since the beginning of 2022 to date, 86,930 laboratory-confirmed cases and 1051 probable cases have been reported worldwide, and of these, 116 were fatal cases.

This outbreak had a certain resonance because, for the first time, most of the cases were registered in countries that have not historically reported monkeypox and without direct or immediate epidemiological links to areas of West or Central Africa where the disease is endemic. 

Indeed, now the monkeypox virus disease is spreading in the American region, followed by African and European countries. In particular, the majority of cases were notified from only 10 countries, which account for 84.5% of the cases reported globally: the United States of America (*n* = 30,091), Brazil (*n* = 10,897), Spain (*n* = 7549), France (*n* = 4144), Colombia (*n* = 4089), the United Kingdom (*n* = 3738), Germany (*n* = 3692), Peru (*n* = 3800), Mexico (*n* = 3956), and Canada (*n* = 1480) [17].

The disease affects all age groups; in endemic countries, children under the age of 16 have historically made up the largest proportion of cases. However, an increase in the incidence of cases in endemic countries has been observed in recent years, with outbreaks reported in all age groups and in more diverse contexts.

In fact, the current data show that, regarding sex, age, and sexual orientation distribution, among cases with known data, a percentage of 96.9% of cases are related to male patients with a median age of 34 years. Furthermore, 79.9% of cases concern males between 18 and 44 years old, 84.8% of cases were diagnosed in men who have sex with men, and in 81.71% of cases, the transmission seems to be related to direct skin and mucosal contact during sexual activities [17,18].

More than half of cases with known HIV status were HIV-positive, and 70.9% of all reported transmission events were due to a sexual encounter. In addition, several cases of syphilis coinfection have been reported recently [18,19].

## 3. Pathogen and Reservoirs of Infection

Monkeypox is a zoonotic infectious disease caused by the Monkeypox virus, a double-stranded DNA virus belonging to the Poxviridae family, genus Orthopoxvirus.

Monkeypox virus is a double-stranded DNA with a genome of around 196,858 base pairs (bp) with around 200 genes [20]. After infecting host cells, the virus lifecycle, despite a DNA genome, occurs in the cytoplasm, where all of the transcription and replication enzymes are encoded from open reading frames (ORFs) of the monkeypox virus genome. These ORFs have more than 90% sequence identity with those of other orthopoxviruses [20]. 

Three categories of viral proteins have been found to be essential to the monkeypox virus lifecycle: viral entry proteins that let the monkeypox virus bind to specific receptors on the membrane surface of the host cells, viral proteins, which are essential for releasing the monkeypox virus copies from the host cells, and proteins involved in the immune modulation of the infected host [21].

There are two genetically distinct clades of the monkeypox virus: the Central African clade (Congo Basin) and the West African clade [22]. The clades are 99.4% identical at the protein level, but they differ in terms of several functionally unique genes, non-functional ORF regions, and additional ORFs. The Congo Basin clade has historically been associated with more serious disease, greater human-to-human transmissibility, and higher lethality [22].

During the current international 2022 outbreak, samples from Portuguese, Belgian, French, German, Dutch, Italian, Spanish, Slovenian, and Brazilian human monkeypox cases were analyzed to perform some preliminary phylogenetic studies of monkeypox virus genomes. The results of these studies showed that the genotype of the monkeypox virus predominant in this current epidemic is that of West Africa [21]. Due to a progressive loss of genes not essential for human pathogenesis, the monkeypox virus in this emergency scenario became a highly adapted virus, causing more serious disease and a rapid and efficient human-to-human transmission [23,24].

Despite the name, a wide range of animals have been identified as being susceptible to the monkeypox virus, which has spread particularly among primates and small rodents, mainly in Africa [25]. In 1977, Breman et al. [26] found anti-OXPV antibodies in primates of the genera *Cercopithecus* and *Colobus* when a serological survey was conducted among non-human primates in West Africa (Côte d’Ivoire, Mali, and the Upper Volta Region). After this first study, others followed that attempted to understand and identify the reservoir or the natural hosts of the monkeypox virus; the findings showed that the main role in the maintenance and circulating of the virus in nature is mainly played by small mammals, even if uncertainties remain about what the natural reservoirs of monkeypox virus are and how the circulation of the virus in nature is maintained. The animal species that seem to be most involved are rope squirrels (*Funisciurus anerythrus* and *F. congicus*) [12], sun squirrels (*Heliosciurus* spp.), non-human primates (primarily *Cercopithecus scanius*) [27], giant pouched rats (*Cricetomys emini*), elephant shrews (*Petrodromus tetradactylus*), domestic pigs (*Sus scrofa*) [12], and dormice [28].

## 4. Transmission and Pathogenesis of Monkeypox Disease

**Figure 1:**
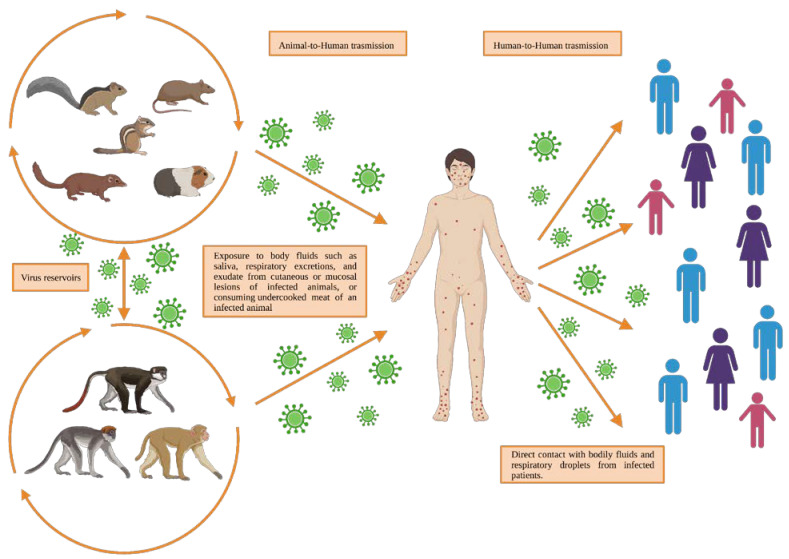
The main route of transmission of the monkeypox virus. Created with BioRender.com.

Human-to-human and animal-to-human are the two monkeypox virus transmission modes that seem to be possible (Figure 1). The exact transmission mechanisms of monkeypox are still unclear; however, animal-to-human transmission is related to direct contact with or exposure to body fluids, such as saliva, respiratory excretions, and exudate from the cutaneous or mucosal lesions of infected animals [29]. Another important exposure source can be viral shedding via feces [30] or consuming the undercooked meat of an infected animal [6,31,32,33].

Often, the precise exposure source can be difficult to determine, especially in regions where contact with animals is common [34] and easier due to poor infrastructure, a lack of adequate health and public hygiene systems, or where people, for lack of resources, feed on small mammals hunted in the surrounding areas [35]. Direct contact with bodily fluids and respiratory droplets from infected patients can result in the human-to-human transmission of the monkeypox infection [6,31,32,33]. Contaminated objects/surfaces, such as bedding, dishes, cutlery and glasses, and sanitary surfaces, are considered to be potential risk factors for viral transmission within the same household. Despite the fact that the World Health Organization (WHO) has not asserted whether the sexual transmission of monkeypox is possible or not, most cases have been reported in males who have sex with males; thus, the transmission can be attributed to close contact, including sexual contact [29]. An infected person remains contagious for the duration of symptomatic illness, usually 2 to 4 weeks. People who have close contact with a case while the disease is contagious, including healthcare workers, domestic partners, and sexual partners, are at an increased risk of infection [36]. Other transmission routes have also been documented, such as that through the placenta, from mother to fetus [37]. Furthermore, the virus has been detected in seminal fluid, in genital and rectal lesions and, in four Italian patients, in feces [38]. However, monkeypox was found in male semen, with a cycle of quantification ranging from 27 to 30, too low to allow for the isolation of live virus. The results, therefore, support the hypothesis that monkeypox can be transmitted sexually, although other transmission possibilities have recently been reported.

The human monkeypox virus disease has a pathogenesis and pathophysiology that are closely related to the transmission cycle of the virus, whether it is of human-to-human or zoonotic origin. The monkeypox virus transmission cycle, in most cases, starts with the virus infecting the exposed mucosa of the oropharyngeal or respiratory tracts of the host. The infection process starts when the virus binds and enters the host cell through interactions of the virus surface proteins with primary attachment receptors (glycosaminoglycans) on the cellular membrane of the host’s cells [39]. After the viral entry phase, the monkeypox virus begins its replication at the level of the inoculation site. Following viral replication, the virus migrates by the lymphomatous route, spreading to the major systemic organs, resulting in primary viraemia. During this phase, the reticuloendothelial system removes the major part of the circulating virus, thus for which the virus is not at all or not very detectable in the blood. In secondary viremia, the virus is released from the infected organs and lymphoid tissues in the blood and reaches the distant lymph nodes and organs, the skin and mucosal epithelium, giving rise to the typical clinical manifestation [29,40,41]. The entire transmission pathway occurs during the incubation period, typically lasting from 5 to 21 days [42] and, in this phase, the patient shows no symptoms, and it is not contagious. 

During the vesicular stage of monkeypox, the histopathological analysis of skin lesions can reveal ballooning degeneration of keratinocytes, prominent spongiosis, dermal edema, and acute inflammation [43]. In the pustule stage, the debris from apoptotic keratinocytes and inflammatory cells are predominant, with few viable keratinocytes, which can be multinucleated or exhibit cytopathic damage such as eosinophilic inclusion bodies, prominent nucleoli, and ground glass chromatin. All keratinocytes within the affected epidermis show the virus in their cytoplasm, as confirmed through immunochemistry, while the lymphocytic infiltrate is mainly composed of T-cells with CD4+ and CD8+ elements [44].

Monkeypox virus infection induces humoral and cellular immune responses in patients who recover from the disease, thereby limiting viral replication and inducing long-term immunity. The humoral immune response after natural infection or vaccination consists of orthopoxvirus-specific IgM and IgG antibodies, which target multiple antigen targets and protect from reinfection or severe disease by long-term persistence of residual IgG-memory B cells [45].

After a natural infection or vaccination, the cellular immune response is marked by a quick increase in activated effector CD4+ and CD8+ T-cells, which typically return to normal levels within 12–20 days from the onset of symptoms [46]. 

The majority of patients harbor T-cells capable of producing a diverse range of inflammatory cytokines, including IFN-γ, IL-1β, IL-6, IL-8, TNF, and MCP-1. The effector CD4+ T-cells contribute to improving the recall and differentiation of B cells, resulting in the production of antibody-secreting cells. The CD8+ T cells have the function of destroying infected macrophages to hinder viral propagation [45].

## 5. Clinical Features and Diagnosis

**Figure 2:**
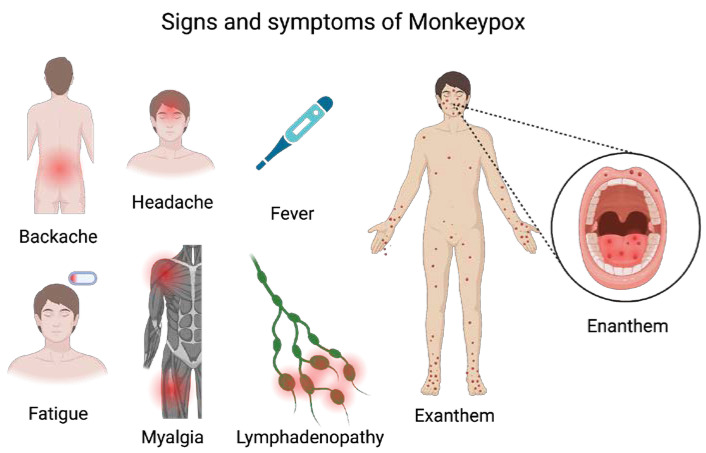
Signs and symptoms of Monkeypox. Created with BioRender.com.

Clinically, the disease (Figure 2) is generally characterized by two symptomatic periods: (1) in 58% of cases, at least one prodromal symptom is present [47]; the prodrome period (0–5 days) is characterized by non-specific symptoms, such as backache, headache, chills, fever, fatigue, myalgia, lethargy, and lymph node swelling [48,49]. Lymphadenopathy can be either generalized or localized. It usually occurs before the appearance of the rash or, in rare cases, at the onset of the rash. Lymphadenopathy, unlike other pox-diseases, is a distinctive feature of monkeypox feature with tender palpable maxillary, cervical and inguinal lymph nodes (1–4 cm), seen in 84% of unvaccinated patients and 54% of vaccinated patients [34]; (2) After three days, in 95% of cases, the fever decreases, and the rash starts spreading centrifugally affecting the face (in 95% of cases) and palms of the hands and soles of the feet (in 75% of cases) [50]. 

The primary areas affected in the current outbreak are the anogenital and perioral regions, with only a small number of lesions appearing on the trunk and limbs. Male patients with genital lesions may experience paraphimosis and necrotic crusts, while rectal lesions can result in proctitis and pain during bowel movements as the initial symptom [18]. 

Some of the reported symptoms related to the rectum included purulent or bloody feces (21%), rectal pain (22%), and rectal bleeding [47].

**Figure 3:**
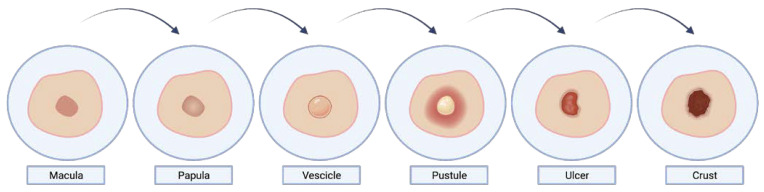
Monkeypox rush evolution. Created with BioRender.com.

Similar to COVID-19, monkeypox disease, in its symptomatological picture, also presents with signs and symptoms in the head and neck region [51,52,53,54,55]. Oral mucous membranes involvement has been reported in 70% of cases [56,57], but also other facial mucosae could be affected, such as conjunctivae (20%), as well as the cornea. The oral lesions typically had the aspect of papules, vesicles, pustules, and ulcers, and could occur in several parts of the oral mucosa, including the lips and tongue, but mainly in the tonsils and are often accompanied by erythema, enanthem, edema, and severe pain along with tonsillar swelling, and sore throat [58]. Oral lesions could lead to problems eating and drinking, which can lead to dehydration and malnutrition. The skin rash (exanthem) is preceded by an oropharyngeal and tongue rash (enanthem) that often goes unnoticed in the preceding 24 h. The skin lesions undergo an evolution from macules and papules to vesicles and pustules that ulcerate and crust which dry up and fall off [59] (Figure 3). The number of lesions varies from a few to several thousand. In severe cases, lesions can coalesce until large sections of skin slough off.

Since the vesicles are well-circumscribed and positioned deep within the dermis, these do not easily rupture (unlike varicella and herpes simplex vesicles). The pustules gradually umbilicate, scab over, and then progressively separate after about 14 days. When the crust drops off, the patient is no longer regarded as contagious. There may be a residual pigment or a depressed scar, especially on the face [34]. 

There are differences in the appearance of the rash between vaccinated and unvaccinated people. People who have been vaccinated have fewer lesions, smaller lesions, and a better depiction of the centrifugal distribution [34].

The signs and symptoms of monkeypox are typically self-limited within 2–4 weeks, but clinical outcomes are usually worse in children, pregnant people, or immunocompromised patients [60], with several possible complications, such as pneumonia, encephalitis, acute kidney injury, visual loss due to corneal infection, myocarditis, hypo-hyperpigmentation, scarring, dehydration (from nausea and vomiting), and secondary bacterial infection, leading to septicemia [6,34,60,61,62]. The majority of recorded deaths have occurred in immunocompromised patients, young adults, and children, with a case–fatality ratio estimated between 1% and 10%. The Central African clade has a case–fatality rate of approximately 11% in Africa in unvaccinated people, while the West African clade causes less severe disease, with a case–fatality rate of less than 4% [35]. Only once the appropriate clinical case definition has been met and the diagnosis has been confirmed through laboratory testing can a final diagnosis be taken into account. When evaluating people with a characteristic rash, a high index of suspicion is recommended, especially when the individual meets one of the epidemiologic criteria. Monkeypox should be included in the differential diagnosis when a patient presents with a sexually transmitted infection (STI)-associated or STI-like rash even if the rash is confined and not (yet) diffuse because the clinical picture may be unusual in this epidemic. Rapid diagnosis is essential for the containment of the virus spread, but often, clinical findings are not accurate enough to achieve a definitive diagnosis. Differential diagnoses include other rash diseases, such as chicken pox, bacterial skin infections, scabies, syphilis, and drug-associated allergies. The presence of lymphadenopathy among the signs of the prodromal phase of the disease may be a clinical characteristic distinguishing monkeypox from chickenpox or smallpox. When faced with a suspected case of monkeypox, it is necessary to collect biological samples from the skin lesions and transport them safely and in accordance with national and international safety requirements to a reference laboratory. Specimens should be stored in a dry, sterile tube and refrigerated. Nucleic acid amplification tests, such as polymerase chain reaction (PCR), are the laboratory tests of choice for confirming the diagnosis, given their specificity and sensitivity. Virus isolation from nasopharynx and oropharynx secretions allows for diagnosis confirmation [61,63,64]. 

## 6. Prevention and Therapy

From the results of several observational studies, it emerges that monkeypox can be prevented via vaccination against smallpox with an efficacy of about 85% [65]. Therefore, people previously vaccinated against smallpox may have a milder disease and a fivefold reduced risk of contracting monkeypox infection compared to unvaccinated people (0.78 vs. 4.05 per 10,000) [66]. Modified vaccinia Ankara (MVA) and ACAM2000 appear to be two potentially effective vaccines in the prevention of monkeypox disease. The type, route of administration, number of doses, injection volume, and time of the immunity peak are summarized in Table 1.

In 2013, the European Medicines Agency (EMA) authorized the MVA-BN vaccine, a third-generation smallpox prevention vaccine based on a modified attenuated vaccine virus (Ankara strain) [67,68]. In 2007, the US FDA approved a live replication-competent smallpox vaccine, ACAM-2000, for smallpox prevention in individuals at high risk of smallpox infection. Since September 2019, the MVA-BN vaccine (sold under the commercial name Imvamune and JYNNEOS) has also been authorized in the USA for the prevention of monkeypox [69]. Vaccination is recommended for laboratory personnel working in contact with the Vaccinia virus or other orthopoxviruses in specialized referral or research facilities. 

The administration of the JYNNEOS vaccine intradermally for patients older than 18 or subcutaneously for patients younger than 18 who are considered to be at high risk for monkeypox infection was approved and licensed by the US Food and Drug Administration on 9 August 2022 [70]. According to CDC recommendations, postexposure vaccination with the MVA vaccine is possible for patients who contact the patient or patient’s materials without personal protection equipment [71] and can prevent the onset of the disease if administrated within 4 days of exposure [72]. Between the 4th and the 14th day, the administration of the MVA vaccine might decrease the severity of the disease [72]. Recently, the ACAM2000 vaccine has been made available for use against monkeypox under an Expanded Access Investigational New Drug (EA-IND) protocol [72]. No data are currently available on the clinical efficacy or effectiveness of JYNNEOS or ACAM2000 for monkeypox disease. It is known that the ACAM2000 vaccine has more known side effects and contraindications, such as myocarditis, pericarditis, and serious vaccine reactions [73]. The treatment of monkeypox is symptomatic and supportive, including the prevention and treatment of secondary bacterial infections. Treatment should be considered for use in people who have severe disease or other conditions requiring hospitalization and the involvement of anatomic areas that might result in serious sequelae that include scarring or strictures; it should also be considered for use in people who are at high risk for severe disease such as immunocompromised patients, pediatric populations, pregnant or breastfeeding people, people with a condition affecting skin integrity [74]. There is currently no approved treatment specifically for monkeypox virus infections [74]. However, several antivirals that were developed for use in smallpox patients may be beneficial against the monkeypox virus [75]. An antiviral known as Tecovirimat, which was developed for smallpox, was authorized by the EMA for the treatment of monkeypox in 2022 but is not yet widely available [76,77]. In vitro and animal studies have also shown efficacy results for Cidofovir [78] and Brincidofovir [79]. The WHO suggests administering vitamin A supplements according to normal recommendations as it plays an important role in the healing of injuries and ocular health. Recently, the CDC has allowed the administration of Vaccinia Immune Globulin (VIG) in Orthopoxviruses outbreaks, such as monkeypox [74]. VIG is obtained from the human plasma of smallpox-vaccinated patients, and the postexposure administration of VIG might be considered in immunosuppressed patients for whom vaccination is contraindicated [80]. The prognosis of the disease depends on multiple factors, including the previous vaccination status, the person’s initial health status, concomitant diseases, and comorbidities.

## 7. Conclusions

The state of alert during the ongoing epidemic derives from the high number of cases of monkeypox disease that have been recorded since May 2022.

It is not yet clear what the distribution of the social and scientific environmental causes of this unexpected increase in cases is and, therefore, an in-depth study using a unified and universal One Health approach (human, environmental and animal) is necessary.

To date, the risk of the sexual transmission of monkeypox underscores the need to raise awareness among the sexually active population to prevent future outbreaks. Preliminary data from available studies suggest that risk factors for contracting monkeypox include being a young male, having sex with other men, having unprotected sex, being HIV positive, and having a history of sexually transmitted infections. Today, prevention campaigns and the control of human monkeypox are the best weapons to prevent infection and stop transmission. Most human monkeypox infections result from a primary animal-to-human transmission, thus, prevention strategies, such as avoiding contact with sick or dead animals and the proper preparation of all foods containing animal meat or parts, should be adopted. Furthermore, close contact with infected people or contaminated materials should be avoided to prevent human-to-human transmission. Furthermore, health workers are at great risk of infection; thus, gloves and other personal protective clothing and equipment should be worn while taking care of patients.

## Figures and Tables

**Table 1:** Vaccines for the Prevention of Monkeypox.

Vaccine	Type	Administration	Doses Number	Injection Dose	Immunity Peak
MVA-BN vaccine	Modified attenuated virus (Ankara strain)	Intradermal (≥18 years) or subcutaneous (<18 years)	2 doses 28 days apart	(≥18 years): subcutaneous 0.5 mL intradermal 0.1 mL (<18 years): subcutaneous 0.5 mL	14 days after 2nd dose
ACAM-2000 vaccine	Live replication-competent virus	Percutaneous (bifurcated needle)	1 dose	0.0025 mL droplet of reconstituted vaccine	28 days after the dose

## Data Availability

Data sharing not applicable.
